# Conspicuous Animals Remain Alert When Under Cover but Do Not Differ in the Temporal Course of Vigilance from Less Conspicuous Species

**DOI:** 10.3390/ani15020214

**Published:** 2025-01-14

**Authors:** Gerhard Hofmann, Claudia Mettke-Hofmann

**Affiliations:** 1 Independent Researcher, Moreton CH46, UK; gerhard@hofmann-photography.de; 2 School of Biological & Environmental Sciences, Liverpool John Moores University, Liverpool L3 3AF, UK

**Keywords:** social vigilance, antipredator behaviour, waterholes, age, Gouldian finch, long-tailed finch

## Abstract

Conspicuousness increases the risk of predation. One strategy to reduce this risk is to increase vigilance. We investigated the frequency of head movements as a measure of vigilance at waterholes in two related songbird species that differed in their conspicuousness: the Gouldian finch and the long-tailed finch. While both species showed a high frequency of head movements (high vigilance) when sitting in an open tree, the more colourful Gouldian finch remained more vigilant than the less conspicuous long-tailed finch when sitting under cover. Moreover, juvenile Gouldian finches had similar vigilance levels than adults; whereas, juveniles of the long-tailed finch were less vigilant, possibly because of staying in the family group. Furthermore, both species showed a similar temporal course of vigilance with a fast decrease in vigilance after landing in the tree, which was affected by group size. Single individuals had much higher vigilance than groups of birds. Finally, vigilance was higher at small waterholes. The results show that conspicuousness, uncertainty when arriving at waterholes, and higher perceived risk at small waterholes increased vigilance.

## Simple Summary

Colourful animals are often more conspicuous than dull animals, which increases their exposure to predators. One way to reduce predation risk could be to be more vigilant. We investigated this in two related songbird species, the colourful Gouldian finch and the less colourful long-tailed finch, at waterholes by measuring the frequency of head movements as a measure of vigilance. Both species showed a high frequency of head movements (high vigilance) when sitting in an open tree where they could be easily spotted. However, the more conspicuous Gouldian finch remained more vigilant when sitting under cover than the long-tailed finch. Moreover, juveniles and adults of the Gouldian finch had similar vigilance levels; whereas, juvenile long-tailed finches were less vigilant than their adult counterparts, possibly because of the juveniles often being in family groups. Both species showed a similar reduction in vigilance shortly after landing in the tree. The reduction in vigilance was affected by group size. This indicates initial uncertainty about the threat they might face, more so when alone. Finally, vigilance was higher at small waterholes, reflecting a higher perceived risk, as compared to larger waterholes.

## 1. Introduction

Animals differ in their conspicuousness from dull to brightly coloured. Higher conspicuousness can result in higher predation (interspecific: [1,2]; intraspecific: [3,4,5,6,7,8,9]), requiring behavioural adaptations, such as higher vigilance [10]. Vigilance is an antipredator behaviour (e.g., [11,12]) but also allows for the monitoring of other individuals (social vigilance, e.g., [12,13,14,15,16,17]). Animals scan their environment to detect any changes that might convey a threat. A high frequency of vigilance (looking up frequently or frequently changing the direction of looking) has been linked to higher vigilance [12,18,19,20,21] and better predator detection [22]. While vigilance generally decreases with group size [11,16,21,23,24,25,26,27,28,29,30] (many eyes hypothesis [31], dilution hypothesis [32]), it can also increase or remain stable with increasing group size due to an increased requirement to monitor group mates (social vigilance; [17,33,34,35]). While a range of factors affecting vigilance have been investigated, such as position in a group (higher vigilance at the periphery: [14,36,37,38]), distance to cover (higher vigilance when further away: [33,39,40,41]), and age (higher vigilance in adults: [20,34,42]), little research has been carried out on how conspicuousness might affect vigilance (but see [43]). The only exception are two studies on songbirds, showing that vigilance was linked to conspicuousness with higher vigilance the more conspicuous the individual [10,44]. Two other studies are on waterfowl with mixed results. More conspicuous mallard (*Anas platyrhynchos*) males in breeding plumage were again more vigilant than less conspicuous males in their nonbreeding plumage [45]. In contrast, higher conspicuousness in male Eurasian wigeons (*Anas penelope*) was attributed to mate guarding rather than higher conspicuousness [46]. Moreover, relatively little is known about the temporal course of vigilance, i.e., how vigilance changes over time, specifically in relation to conspicuousness.

Vigilance is not static but should adjust to the spatiotemporal fluctuation of risk. For example, risk can change diurnally (linked to activity of main predators; [47,48,49]) or seasonally [34,50,51,52,53]. Lima and Bednekoff 1999 [54] suggested that animals should adjust antipredator behaviour to the temporal fluctuation of risk (risk allocation hypothesis), which has been confirmed in many studies [24,41,49,55,56,57,58,59]). Moreover, Sirot, and Pays (2011) [60] suggested that vigilance changes over time, predicting that, on arrival at a location, vigilance should be high, as the newcomer faces uncertainty regarding threats around. Over time, the individual collects information about potential threats reducing uncertainty. This then results in reduced vigilance. This has been confirmed in a range of mammal and bird species [36,61,62,63,64,65].

Additionally, not every location carries equal risk. For example, areas vary in predator presence/absence [21,66,67,68,69], frequency of encounters with predators [38,70], available cover [39,41,59,71,72,73], etc., with associated higher vigilance when risk is higher. Furthermore, places that are regularly visited by prey (e.g., waterholes, carcasses, fruiting trees) attract predators and require higher vigilance [74]. For example, most animals visit waterholes at least once a day, which makes such places particularly risky to visit [23,47], as ambush predators can wait [75]. Similar risks arise when visiting fruiting trees or carcasses over a period of time [40,76]. To reduce this risk, animals often prefer waterholes that provide cover for their own protection [77,78] but also allow a relatively free view to spot predators early on [78]. Furthermore, animals increase vigilance around waterholes [13], more so the less cover they have [79]. Likewise, the lack of cover also increases vigilance at carcasses [40], fruiting trees [76], or feeders [10]. Like at other locations, vigilance decreases with increasing group size at waterholes [79,80,81] and increases with higher perceived risk [74,80]. Finally, waterhole size affects vigilance, with higher vigilance at small waterholes likely reflecting higher risks [79].

Most research at waterholes has been carried out on ungulates [47,74,80,81] (but see [79,82]), despite nearly every species across taxa visiting waterholes on a regular basis. Furthermore, little is known about the temporal dynamics of vigilance at waterholes and how it is affected by individual characteristics and environmental factors. Here, we investigated the temporal dynamics of vigilance at waterholes in relation to conspicuousness, cover, and group size in two estrildian finches: the Gouldian finch (*Chloebia gouldiae*) and the long-tailed finch (*Poephila acuticauda*). Both species co-occur in North Australia’s savannah grassland but differ in their plumage conspicuousness, with the Gouldian finch being much more colourful than the long-tailed finch. Gouldian finches have been shown to be more vigilant when perched in open trees without cover, as compared to when in trees with cover, when alone, and at small waterholes [79]. No vigilance studies have been conducted on long-tailed finches so far. We predicted the following:

(A) Gouldian finches are more vigilant than long-tailed finches at waterholes to account for their higher conspicuousness;
(B) Both species reduce their vigilance over time, as they assess threats;
(C) Cover, increasing group size, and larger waterholes would decrease vigilance.

## 2. Materials and Methods

### 2.1. Study Species and Location

Both species are endemic to North Australia and have similar habitat requirements. The Gouldian finch is a habitat and diet specialist, inhabiting tropical savannah grassland [83] and feeding largely on sorghum grasses on the ground [84]. They are nomadic during the nonbreeding season, following food availability [83]. The species is listed as endangered by the Australian Department of Environment, Parks, and Water Security [85]. Gouldian finches are brightly coloured with a green back, yellow belly, and purple breast. They occur in three distinct head colour morphs with 70% black-headed birds, 30% red-headed birds, and less than 1% yellow-headed birds in the same population [86]. The colour polymorphism is present in both sexes. Additionally, Gouldian finches are sexually dimorphic, with males overall having a brighter plumage and longer central tail feathers. Juveniles are uniformly grey green in colour [87].

The long-tailed finch occurs sympatrically with the Gouldian finch but has a broader habitat range, including grassy bushland and pandanus savanna, and a diet ranging from grassy seeds to insects [87]. The species is resident but shows local movements during the dry season [87]. Long-tailed finches are grey–brown in colour, with the chin to upper breast being black. They have a yellow beak (western subspecies *P. a. acuticauda*) and long central tail feathers. Sexes look largely alike. Juveniles are overall paler with a greyish throat and black bill but look otherwise the same [84]. The two species are competitors over nest cavities with long-tailed finches usually outcompeting Gouldian finches [88]. However, Gouldian finches also use long-tailed finches to assess risk at waterholes [89].

The study was carried out during the dry, nonbreeding season between July and August around waterholes in the Kimberley region of Western Australia. Data were collected between Wyndham in the West (15°29′08.3″ S 128°07′14.9″ E) and Lake Argyle in the East (16°05′55.4″ S 128°42′17.1″ E), largely along the Great Northern and Victoria Highway. The only location off the Highway was in the El Questro resort (16°00′29″ S 127°58′50″ E). The habitat in the study area and around all waterholes was characterised by open eucalyptus woodland consisting of a mixture of eucalyptus (*Eucalyptus* spp.), bloodwood (*Corymbia* spp.), and boab (*Adansonia gregorii*) trees with annual sorghum grass (*Sorghum* spp.) on the ground.

Seven waterholes were sampled (six for long-tailed finches due to logistic reasons; Table 1). Waterholes were on median 12.5 km (quartile ranges 6.8–47.0 km) apart but at least 1.5 km (one location; for more details see [76]). The two closest locations were separated by a ridge and visited by different individuals, as numbers and compositions differed between the two sites. It is, therefore, assumed that waterholes are independent sampling units. Waterholes were remnants of creeks or man-made and ranged in size from <1 m^2^ (small) to stretches of creeks of 100 m lengths and 10 m width (large; Table 1; for a detailed description see further down). All waterholes had a mixture of open trees without leaves (boabs or dead trees) and trees with foliage providing cover. Brown falcons (*Falco berigora*) and Brown goshawks (*Accipiter fasciatus*), two of the main avian predators there, were present at all waterholes and initiated attacks on a daily basis. Waterholes were selected based on long-term use (pers. comm. Gary Fitt), local knowledge, and finding new waterholes used by Gouldian finches.

### 2.2. Data Collection

Data collection occurred between 5:30 and 10:00 am every morning. The observer (CMH) positioned herself about 10–15 m away from the waterhole hidden in vegetation. Birds were observed with binoculars and data recorded on a Dictaphone (Sony IC Recorder ICD-PX440, Sony, Tokyo, Japan). Recording started when an individual of one of the two species landed in a tree near the waterhole. As the Gouldian finch was much rarer than the long-tailed finch, precedence was given to the Gouldian finch whenever they were present and data on long-tailed finches were only collected when no Gouldian finches were present. However, the data collection of long-tailed finches often occurred just before or after Gouldian finches were sampled, i.e., both species were equally distributed across the sampling period every day. Species ID, age, sex (Gouldian finch only), head colour (Gouldian finch only), location (open, medium, or dense, see below), and number of other individuals of the same species [24,74] in the same or neighbouring tree were recorded before commencing the collection of vigilance data. With the Dictaphone running, every head movement of the focal bird was counted and quietly spoken into the Dictaphone. A head movement could be vertical or horizontal, as every change in the head’s position changes the eye’s view and brings a different part of the environment into focus [90]. Blocks of 20 head movements were counted before counting started again, until ideally three blocks of head movements were recorded. Shorter blocks were recorded in case the bird changed cover or flew away. The three blocks of 20 head movements were recorded to allow analysis of the temporal course of vigilance. Sixty head movements approximated to about one minute of vigilance data comparable to other vigilance studies [23,91,92,93]. After the recording of three blocks of head movements, the number of birds present of this species was assessed again. When other individuals of this species were present, a different individual was chosen and counting started again. Each location was observed two to seven times, depending on the number of birds (specifically Gouldian finches) visiting the waterhole. Visits were on average 3.8 days ± 1.9 days (mean ± SE) apart.

Gouldian finches visit waterholes once a day, typically in the morning [94]. Therefore, we assumed that we sampled different birds on a given day supported by different group sizes and group compositions. Likewise, group composition and group sizes varied across days indicating different groups visiting the waterholes. To reduce the risk of resampling, we observed birds on fewer days when numbers at a waterhole were overall small. The routine of waterhole visits of long-tailed finches was unknown, but given the large numbers of this species visiting each waterhole, it is unlikely that we resampled the same birds.

### 2.3. Data Analysis

All analyses were conducted with IBM SPSS Statistics v 29.0.1.0, and the data are available in Table S1. The overall sample size was 517, comprising of 302 Gouldian finches and 215 long-tailed finches with at least two blocks of head movements (89 Gouldian finches and 76 long-tailed finches with the remaining individuals (213 and 139, respectively) contributing three blocks) recorded to allow for temporal analyses. We calculated the frequency of head movements by extracting the time it took to make 20 or fewer but at least 10 (in case the bird left before the last block could be completed; 45% of cases) head movements and divided the number of head movements by the corresponding time. Data were square-root-transformed to obtain normally distributed data.

Generalised linear mixed models were used for analysis, with individuals nested within a location and block as a repeated measure, reflecting the temporal course of vigilance. An identity link function was used. Degrees of freedom were calculated with the Satterthwaite approximations due to unequal sample sizes. The main factors were waterhole size (3 levels; small < 1 m^2^–medium 1–5 m^2^–large > 10 m^2^) and location (2 levels; open tree without leaves and dense tree with leaves combining medium and dense tree cover) as ecological factors and species (2 levels; Gouldian finch and long-tailed finch), number of birds (5 levels; 1 = alone, 2 = 2 birds, 3 = 3–4 birds, 4 = 5–7 birds, 5 = >7 birds), and age (2 levels; juvenile, adult) as social factors. For group sizes, we tried to retain as much information by also considering sample sizes within levels. Single birds are likely more alert than when associating with others. Levels two to four reflect socially meaningful groupings, with two birds often being pairs and three to four birds (level 3) and five to seven birds (level 4) not being unusual for family groups. These levels might be perceived differently than larger groups of unknown birds (level 5). Two-way interactions included species with all other factors (waterhole size, location, number of birds, age) to test for differences in vigilance between species. Likewise, the interaction term block with all other factors (waterhole size, location, species, number of birds, age) was included to test whether these factors affected the temporal course of vigilance, i.e., whether the frequency of head movements changed over time and in relation to these factors. The variable ‘individual’ was used as a random factor to account for repeated testing. Posthoc comparisons were carried out with sequential Sidak to adjust for multiple comparisons. Nonsignificant terms were removed starting with the least significant two-way interactions.

## 3. Results

**Figure 1:**
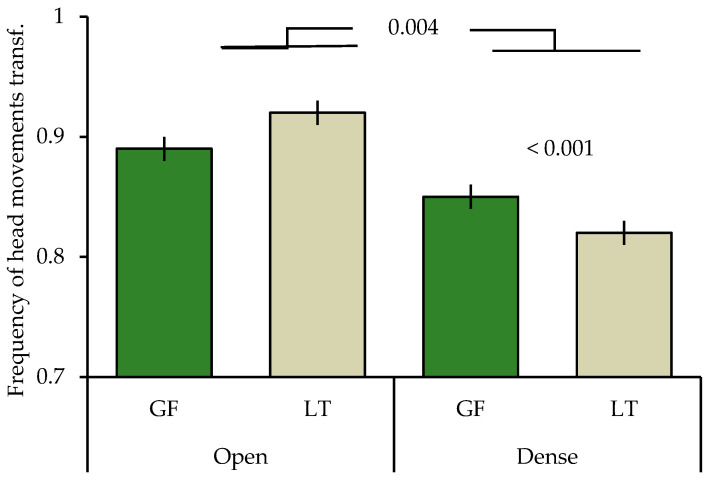
Mean ± SE of frequency of head movements of Gouldian finches and long-tailed finches in relation to cover at waterholes in the Kimberley region, WA, Australia. GF: Gouldian finch; LT: long-tailed finch; transf: transformed. Numbers above the column indicate significant levels.

The final model included all main factors and three two-way factors (Table 2). Gouldian finches and long-tailed finches differed in the frequency of head movements depending on the cover they were sitting in (species × location; Table 2) with Gouldian finches having a higher frequency of head movements when sitting in trees with dense cover, as compared to long-tailed finches (posthoc: t = 3409, *p* < 0.001). The frequency of head movements did not differ between the two species when sitting in open trees (t = 0.530, *p* = 0.569). Overall, both species made more head movements when in open trees, as compared to when in dense trees (t = 2.858, *p* = 0.004; Figure 1).

**Figure 2:**
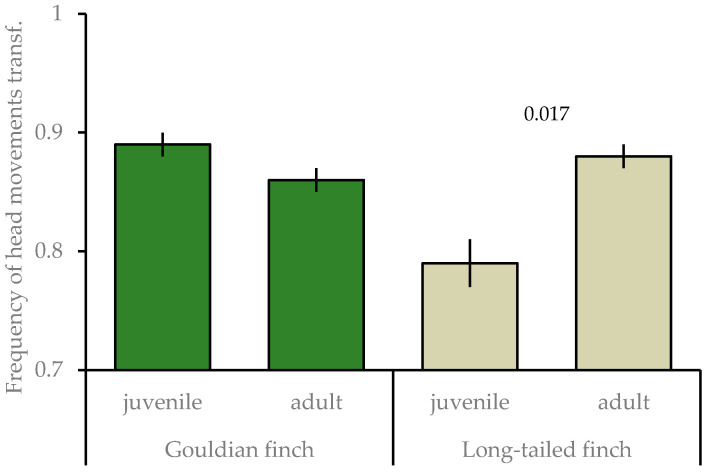
Mean ± SE of frequency of head movements of juvenile and adult Gouldian finches and long-tailed finches at waterholes in the Kimberley region, WA, Australia. Numbers above the column indicate significant levels; transf: transformed.

Furthermore, the interaction species × age was significant (Table 2), with juvenile long-tailed finches having a lower frequency of head movements than adults (t = −2.399, *p* = 0.017); whereas, age classes did not differ in the Gouldian finch (t = 0.862, *p* = 0.389; Figure 2). Any other species differences were not significant and dropped out of the final model.

**Figure 3:**
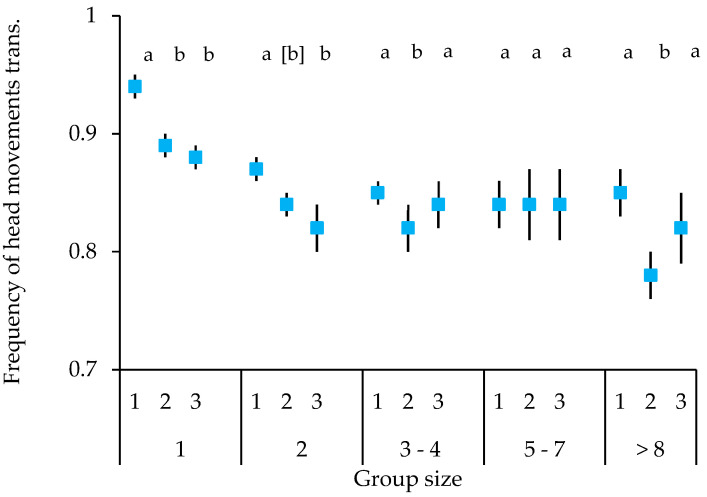
Mean ± SE of frequency of head movements over time (blocks) in relation to group size for Gouldian finches and long-tailed finches combined at waterholes in the Kimberley region, WA, Australia. The upper numbers on the x-axis represent the vigilance blocks 1–3, the lower numbers the group size; different letters indicate significant differences in relation to the first block in each group size; brackets indicate trend; transf: transformed.

Both species showed a similar temporal course of vigilance. Overall, the frequency of head movements decreased over time. However, this differed, depending on the number of birds present (block × number of birds interaction; Figure 3). The highest frequency of head movements overall was observed in the first block when the birds were alone. Frequencies were then significantly decreased in the second and third block (block 1 vs. 2: t = −5.428, *p* < 0.001; block 1 vs. 3: t = −5.893, *p* < 0.001). This picture was largely repeated in the other group sizes, but with the frequency of head movements clearly lower than when alone. Specifically, the frequency of head movements was significantly higher in the first, as compared to the second block in group sizes of two (trend only t = −1.765, *p* = 0.078), three to four birds (t = −2.142, *p* = 0.033), and more than eight birds (t = −3.486, *p* < 0.001). The results were more variable when it came to the third block, with only group sizes of one and two birds showing a significantly lower frequency of head movements, as compared to the first block (group size 2, block 1 vs. 3: t = −2.654, *p* = 0.008).

**Figure 4:**
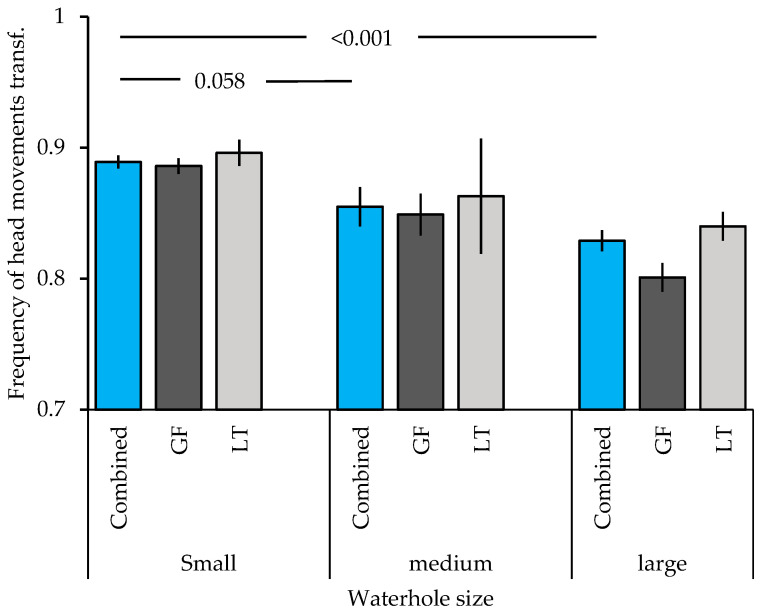
Frequency of head movements (mean ± SE) of Gouldian finches and long-tailed finches at differently sized waterholes in the Kimberley region, WA, Australia. The blue columns represent the frequency of head movements of both species combined, the dark grey represent Gouldian finches (GF), and the light grey one long-tailed finches (LT). Numbers above the column indicate significant levels; transf: transformed.

Finally, waterhole size affected the frequency of head movements across both species with a higher frequency at small waterholes, as compared to medium sized waterholes (trend t = −1.903, *p* = 0.058) and large waterholes (t = −4.435, *p* < 0.001; Figure 4).

## 4. Discussion

Gouldian finches and long-tailed finches differed in their vigilance with respect to the cover they were in. While both species showed high vigilance in open trees, long-tailed finches had lower vigilance levels than the Gouldian finches when in dense trees. Furthermore, juvenile long-tailed finches were less vigilant than adults; whereas, no significant differences were found between age classes in the Gouldian finch. The temporal course of vigilance did not differ between species, but the frequency of head movements was generally higher for the first twenty head movements (first block), as compared to the following twenty head movements (second block), and was highest when alone in the tree, as compared to other group sizes. Finally, vigilance was higher at small waterholes.

Vigilance was high when birds were sitting in open trees, reflecting the perceived higher risk when in an exposed position, as compared to perching in a tree with leaves providing cover supporting the risk allocation hypothesis [54]. This corroborates other studies that found higher vigilance in more threatening situations, e.g., when away from cover [39,41,59,71,72,73,79]. In this high-risk situation, any potential species differences were possibly overruled by the need to be alert, which does not allow for a lot of variation in vigilance. Similar results were found in ungulates. At waterhole sites with additional human hunting activity, i.e., higher risks, no differences in vigilance were found between three ungulate species; whereas, vigilance differed among the same species at waterholes in areas without additional human hunting pressure [92]. The latter was also observed in our study as species differences occurred when under cover, i.e., experiencing a less threatening environment. Gouldian finches were significantly more vigilant than long-tailed finches when sitting under cover, which in part, supports our first prediction. The colourful Gouldian finches might stick out, even when sitting among leaves, making them more conspicuous and an easier target for predators. Higher vigilance has also been found in the more conspicuous sex among sexually dichromatic bird species [10,44,45]. However, the current study seems to be the first one to show differences in vigilance linked to conspicuousness between species.

The two species also differed in their vigilance in relation to age. Juvenile long-tailed finches were less vigilant than adults; whereas, no significant differences were found in the Gouldian finch. Young animals are often found to be less vigilant, as they might face a trade-off with foraging and/or are less experienced in recognising threats [20,34,42,95]. Juvenile Gouldian finches did not follow this pattern and, while not significant, tended to be more vigilant than adults. This is surprising and not linked to conspicuousness, as the young are inconspicuous grey–green. However, it resembles findings from earlier studies with juvenile Gouldian finches being more vigilant than adult males [79]. At this time of year deep into the dry season, most family groups have broken up and juvenile Gouldian finches move around independently or in small juvenile groups. This might increase risks due to their inexperience, resulting in higher vigilance [15]. Alternatively, Gouldian finches might develop adult vigilance patterns early on as an adaptation to breaking up family groups [15]. Long-tailed finches, in contrast, were still in their family groups, as two adults often arrived with juveniles together. This provides more protection and learning opportunities from adults. Reliance on adult vigilance has been shown in other studies [34,96].

Vigilance decreased over time, following a similar time course in both species supporting the second hypothesis predicting that vigilance would decrease over time as individuals assess threats. When arriving in a tree, birds must assess the surroundings and scan for potential predators, as well as registering other birds in the tree. Over time, uncertainty decreases, allowing for lower vigilance [60]. Similar changes in vigilance over time have been found in Barnacle geese (*Branta leucopsis*; [61]) and several gull species (*Larus spec*; [64]) but also in mammals [36,63]. The decrease was particularly prevalent from the first to the second block; whereas, the third block was much more variable. This might be due to the birds preparing to fly down to the waterhole, which requires looking for a place to land and potentially checking on other birds who might fly down as well to avoid collisions, coordinate movement, and/or decide with whom to fly down, ultimately increasing vigilance.

Interestingly, vigilance decreased within a very short period of time. Given that three blocks of vigilance usually take roughly 60 s to complete [79], the observed changes in vigilance occurred within 20 s or less. This means that the initial assessment of the situation was very fast, and without discovering any threats, birds decreased vigilance in less than a minute. This is the fastest adjustment in vigilance observed so far. This might be predominantly due to the time scale chosen in other studies which ranged from 1 min segments [63,64] to 3 min segments [36], as well as 10 min [61] and 30 min sessions [62]. However, in the study by Terhune and Brilliant [36], seals hauled up for resting remained highly vigilant for the first two 3 min segments and only then reduced their vigilance indicating a much longer period of high vigilance. In contrast, vigilance decreased from the first to the second minute segment in several gull species when loafing [64] and in grey squirrels (*Sciurus carolinensis*) in an experimental arena [63] resembling our findings. Future studies might use shorter segments to identify the temporal course of vigilance more precisely.

The temporal decrease in vigilance was strongly affected by group size with a much steeper decrease when birds were alone, as compared to all other group sizes. When birds arrived alone, their initial vigilance was by far the highest, but this then decreased quickly; although, it remained higher than in any other group size. This reflects the vulnerability of single individuals and the need to be alert (many eyes hypothesis [31], dilution effect [32]) and has been observed across species (e.g., [11,25,27]). The strong decrease in vigilance when alone might have two reasons. Firstly, birds might have assessed the situation quickly, as there was a clear view, and they primarily had to pay attention to potential predators, suitable hiding places, and what was going on around the waterhole. Twenty seconds might be enough to establish that there was no immediate danger. Birds might then have subsequently returned to their usual vigilance frequency to keep control of the situation, including visually examining the waterhole for the best place to drink, which would require looking in one direction for longer [97]. This corresponds with the two vigilance strategies proposed by Fernandez-Juricic [97]: visual search, characterised by a high frequency of head movements to cover a large area in a short period of time to discover any threats, and visual tracking, allowing the collection of information, such as estimating distance to target, etc. Secondly, vigilance might have decreased rapidly due to high costs of vigilance [98]. However, this seems less likely, as the birds were sitting in the tree and would not lose any foraging time by turning their head more often. When birds were in groups, the pattern repeated but at a lower vigilance level and with a less steep decline, possibly due to starting with reduced vigilance in the first place. It should be noted that vigilance dropped markedly in the second block when more than eight birds (usually more than 20) were present, indicating that larger flocks might provide additional protection.

Finally, waterhole size affected overall vigilance. Both species showed higher vigilance when at smaller waterholes in support of the third prediction. Small waterholes might be perceived as more dangerous, as the birds have fewer options where to land and drink, which makes them more vulnerable to predation. Moreover, competition might arise among birds where to land, which would increase social vigilance [17,33,34,35] to decide when to go down and with whom. Higher vigilance at smaller waterholes in Gouldian finches has been shown in an earlier study [79]. However, this study expands this to the long-tailed finch, too. This indicates that higher vigilance at small waterholes might be a more widespread phenomenon with potentially long-term consequences. With global warming, the frequency and duration of heat waves is increasing globally [78]. Consequently, birds will encounter small waterholes more often and for longer periods of time during the dry season. When higher vigilance is linked to the increased perceived threat of predation and competition with others over extended periods, then this might induce stress. More research is needed into how waterhole size might affect the wellbeing of birds.

## 5. Conclusions

Gouldian finches maintained higher vigilance than long-tailed finches when sitting under cover, potentially due to their higher conspicuousness. Likewise, juvenile Gouldian finches showed similar vigilance levels or higher than adults; whereas, juvenile long-tailed finches had lower vigilance levels than adults, which is likely linked to their different social structure. Both species showed a similar time course of vigilance, with high initial vigilance to assess any threats followed by a reduction in vigilance as uncertainty decreased with a marked effect of group size on overall vigilance. Finally, both species were more vigilant at small waterholes, reflecting higher perceived threats and increased competition.

## Figures and Tables

**Table 1:** Waterhole characteristics and sample sizes of Gouldian finches and long-tailed finches in the Kimberley region, Western Australia, to investigate vigilance.

Location	Natural or Man-Made	Waterhole Size	Number of Focal Birds GF *	Number of Focal Birds LT *
1	Natural	Small	208 (149)	- **
2	Man-made	Large	9 (4)	24 (19)
3	Natural	Small	56 (36)	97 (70)
4	Natural	Medium	35 (30)	9 (8)
5	Natural	Large	17 (15)	31 (23)
6	Natural	Large	63 (40)	59 (47)
7	Man-made	Small	32 (28)	58 (48)
TOTAL			420 (302)	278 (215)

* Numbers outside brackets are the total number of birds observed; numbers in brackets represent the birds that had two or three vigilance blocks and were used for the analysis. ** Long-tailed finches were present but were not sampled due to logistic reasons.

**Table 2:** Outcome of generalized linear mixed model investigating the temporal course of vigilance in Gouldian finches and long-tailed finches at waterholes in the Kimberley region, WA, Australia.

Variable	F-Value	DF 1	DF 2	*p*-Value
Corrected model	9.458	21	720	<0.001
Waterhole size	10.632	2	516	<0.001
Location (open-dense)	31.288	1	514	<0.001
Species	0.735	1	515	0.392
Group size	8.948	4	711	<0.001
Age	3.340	1	512	0.068
Species × location	4.026	1	513	0.045
Species × age	6.344	1	516	0.012
Group size × block	7.124	10	598	<0.001

DF: degrees of freedom.

## Data Availability

All data are available in the Supplementary Materials part of this paper.

## Supplementary Materials

The following supporting information can be downloaded at: https://www.mdpi.com/article/10.3390/ani15020214/s1, Table S1: Data file.

## References

[1] Stuart-Fox D.M., Moussalli A., Marshall N.J., Owens I.P.F. (2003). Conspicuous males suffer higher predation risk: Visual modelling and experimental evidence from lizards. Anim. Behav..

[2] Simpson R.K., Mistakidis A.F., Doucet S.M. (2020). Natural and sexual selection shape the evolution of colour and conspicuousness in North American wood warblers (Parulidae). Biol. J. Linn. Soc..

[3] Montgomerie R., Lyon B., Holder K. (2001). Dirty ptarmigan: Behavioral modification of conspicuous male plumage. Behav. Ecol..

[4] Husak J.F., Macedonia J.M., Fox S.F., Sauceda R.C. (2006). Predation cost of conspicuous male coloration in collared lizards (*Crotaphytus collaris*): An Experimental test using clay-covered model lizards. Ethology.

[5] Nasri I., Hamza F., Belliure J., Selmi S. (2018). Tail conspicuousness and antipredatory behaviour in Bosk’s fringe-toed lizard (*Acanthodactylus boskianus*). Ethol. Ecol. Evol..

[6] Szopa-Comley A.W., Donald W.G., Ioannou C.C. (2020). Predator personality and prey detection: Inter-individual variation in responses to cryptic and conspicuous prey. Behav. Ecol. Sociobiol..

[7] Valkonen J.K., Vakkila A., Pesari S., Tuominen L., Mappes J. (2020). Protective coloration of European vipers throughout the predation sequence. Anim. Behav..

[8] Agan J., Macedonia J.M., Grindstaff J.L., Fox S.F. (2024). Orange ornamentation increases sex-specific conspicuousness of juvenile males to conspecifics and predators. Biol. J. Linn. Soc..

[9] Poloni R., Dhennin M., Mappes J., Joron M., Nokelainen O. (2024). Exploring polymorphism in a palatable prey: Predation risk and frequency dependence in relation to distinct levels of conspicuousness. Evol. Lett..

[10] Pascual J., Senar J.C., Domenech J. (2014). Plumage brightness, vigilance, escape potential, and predation risk in male and female Eurasian Siskins (*Spinus spinus*). Auk.

[11] Watson M., Aebischer N.J., Cresswell W. (2007). Vigilance and fitness in grey partridges *Perdix perdix*: The effects of group size and foraging-vigilance trade-offs on predation mortality. J. Anim. Ecol..

[12] Pascual J., Senar J.C. (2013). Differential effects of predation risk and competition over vigilance variables and feeding success in Eurasian siskins (*Carduelis spinus*). Behaviour.

[13] Rose L.M., Fedigan L.M. (1995). Vigilance in white-faced capuchins, *Cebus capucinus*, in Costa Rica. Anim. Behav..

[14] Klose S.M., Welbergen J.A., Goldizen A.W., Kalko E.K.V. (2009). Spatio-temporal vigilance architecture of an Australian flying-fox colony. Behav. Ecol. Sociobiol..

[15] Beauchamp G. (2018). The effect of age on vigilance: A longitudinal study with a precocial species. Behaviour.

[16] Fattorini N., Lovari S., Brunetti C., Baruzzi C., Cotza A., Macchi E. (2018). Age, seasonality, and correlates of aggression in female Apennine chamois. Behav. Ecol. Sociobiol..

[17] Zhao J.-M., Lyu N., Sun Y.-H., Zhou L.-Z. (2019). Number of neighbors instead of group size significantly affects individual vigilance levels in large animal aggregations. J. Avian. Biol..

[18] Mathot K.J., van den Hout P.J., Piersma T., Kempenaers B., Reale D., Dingemanse N.J. (2011). Disentangling the roles of frequency-vs. state-dependence in generating individual differences in behavioural plasticity. Ecol. Lett..

[19] Esattore B., Rossic A.C., Bazzonic F., Riggioc C., Oliveirac R., Leggieroc I., Ferretti F. (2020). Erent time, head up: Multiple antipredator responses to a recolonizing apex predator. Curr. Zool..

[20] Kong D., Moeller A.P., Zhang Y. (2021). Disturbance and predation risk influence vigilance synchrony of black-necked cranes *Grus nigricollis*, but not as strongly as expected. Ecol. Evol..

[21] Reimers E., Eftestol S., Colman J.E. (2021). Vigilance in reindeer (*Rangifer tarandus*); evolutionary history, predation and human interference. Polar. Biol..

[22] Cresswell W., Quinn J.L., Whittingham M.J., Butler S. (2003). Good foragers can also be good at detecting predators. Proc. R. Soc. B.

[23] Burger J., Gochfeld M. (1992). Effect of group size on vigilance while drinking in the coati, *Nasua narica* in Costa Rica. Anim. Behav..

[24] Creel S., Schuette P., Christianson D. (2014). Effects of predation risk on group size, vigilance, and foraging behavior in an African ungulate community. Behav. Ecol..

[25] Ye Y., Jiang Y., Hu C., Liu Y., Qing B., Wang C. (2017). What makes a tactile forager join mixed-species flocks? A case study with the endangered Crested Ibis (*Nipponia nippon*). Auk.

[26] Pecorella I., Fattorini N., Macchi E., Ferretti F. (2019). Sex/age differences in foraging, vigilance and alertness in a social herbivore. Acta Ethol..

[27] Saltz D., Berger-Tal O., Motro U., Shkedy Y., Raanan N. (2019). Conservation implications of habituation in Nubian ibex in response to ecotourism. Anim. Cons.

[28] Taraborelli P., Moreno P., Mosca Torres M.E. (2019). Are there different vigilance strategies between types of social units in *Lama guanicoe*?. Behav. Proc..

[29] Scheijen C.P.J., van der Merwe S., Ganswindt A., Deacon F. (2021). Anthropogenic influences on distance travelled and vigilance behavior and stress-related endocrine correlates in free-roaming giraffes. Animals.

[30] Olson E.R., van Deelen T.R. (2024). Competition and sex-age class alter the effects of group size on vigilance in white-tailed deer *Odocoileus virginianu*. Acta Ethol..

[31] Fairbanks B., Dobson F.S. (2006). Mechanisms of the group-size effect on vigilance in Columbian ground squirrels: Dilution versus detection. Anim. Behav..

[32] Bertram B.C.R., Krebs J.R., Davies N.B. (1978). Living in groups: Predators and prey. Behavioural Ecology: An Evolutionary Approach.

[33] Aviles J.M., Bednekoff P.A. (2007). How do vigilance and feeding by common cranes *Grus grus* depend on age, habitat, and flock size?. J. Avian Biol..

[34] Blank D.A. (2018). Vigilance, staring and escape running in antipredator behavior of goitered gazelle. Behav. Proc..

[35] Bernardi-Gomez C., Valdivieso-Cortadella S., Llorente M., Aureli F., Amici F. (2023). Vigilance has mainly a social function in a wild group of spider monkeys (*Ateles geoffroyi*). Am. J. Primatol..

[36] Terhune J.M., Brilliant S.W. (1996). Harbour seal vigilance decreases over time since haul out. Anim. Behav..

[37] Fernandez-Juricic E., Beauchamp G., Treminio R., Hoover M. (2011). Making heads turn: Association between head movements during vigilance and perceived predation risk in brown-headed cowbird flocks. Anim. Behav..

[38] Robinson B., Merrill E.H. (2013). Foraging vigilance trade-offs in a partially migratory population: Comparing migrants and residents on a sympatric range. Anim. Behav..

[39] Coolen I., Giraldeau L.-A. (2003). Incompatibility between antipredatory vigilance and scrounger tactic in nutmeg mannikins, *Lonchura punctulate*. Anim. Behav..

[40] Wikenros C., Stahlberg S., SAND H. (2014). Feeding under high risk of intraguild predation: Vigilance patterns of two medium-sized generalist predators. J. Mammal..

[41] Dannock R.J., Pays O., Renaud P.-C., Marond M., Goldizen A.W. (2019). Assessing blue wildebeests’ vigilance, grouping and foraging responses to perceived predation risk using playback experiments. Behav. Proc..

[42] Arenz C.L., Leger D.W. (2000). Antipredator vigilance of juvenile and adult thirteen-lined ground squirrels and the role of nutritional need. Anim. Behav..

[43] Beauchamp G. (2015). Animal Vigilance: Monitoring Predators and Competitors.

[44] Diniz P. (2011). Sex-dependent foraging effort and vigilance in coal-crested finches, *Charitospiza eucosma* (Aves: Emberizidae) during the breeding season: Evidence of female-biased predation?. Zoologia.

[45] Lendrem D.W. (1983). Sleeping and vigilance in birds. 1. Field observations of the Mallard (*Anas platyrhynchos*). Anim. Behav..

[46] Guillemain M., Caldow R.W.G., Hodder K.H., Goss-Custard J.D. (2003). Increased vigilance of paired males in sexually dimorphic species: Distinguishing between alternative explanations in wintering Eurasian wigeon. Behav. Ecol..

[47] Crosmary W.-G., Valeixa M., Fritza H., Madzikandad H., Côté S.D. (2012). African ungulates and their drinking problems: Hunting and predation risks constrain access to water. Anim. Behav..

[48] Lashley M.A., Chitwood C., Biggerstaff M.T., Morina D.L., Moorman C.E., DePerno C.S. (2014). White-tailed deer vigilance: The influence of social and environmental factors. PLoS ONE.

[49] Eccard J.A., Meißner J.K., Heurich M. (2017). European Roe Deer Increase Vigilance When Faced with Immediate Predation Risk by Eurasian Lynx. Ethology.

[50] Vasquez R.A., Ebensperger L.A., Bozinovic F. (2002). The influence of habitat on travel speed, intermittent locomotion, and vigilance in a diurnal rodent. Behav. Ecol..

[51] Flamand A., Rebout N., Bordes C., Guinnefollau L., Berges M., Ajak F., Siutz C., Petit O. (2019). Hamsters in the city: A study on the behaviour of a population of common hamsters (*Cricetus cricetus*) in urban environment. PLoS ONE.

[52] Hume G., Brunton E., Burnett S. (2019). Eastern grey kangaroo (*Macropus giganteus*) vigilance behaviour varies between human-modified and natural environments. Animals.

[53] Han L., Blank D., Wang M., Yanget W. (2020). Vigilance behaviour in Siberian ibex (*Capra sibirica*): Effect of group size, group type, sex and age. Behav. Proc..

[54] Lima S.L., Bednekoff P.A. (1999). Temporal variation in danger drives antipredator behavior: The predation risk allocation hypothesis. Am. Nat..

[55] Dacier A., Maia R., Agustinho D.P., Barros M. (2006). Rapid habituation of scan behavior in captive marmosets following brief predator encounters. Behav. Proc..

[56] Poudel B.S., Spooner P.G., Matthews A. (2016). Behavioural changes in marmots in relation to livestock grazing disturbance: An experimental test. Eur. J. Wildl. Res..

[57] Costelloe B.R., Rubenstein D.I. (2018). Temporal structuring of vigilance behaviour by female Thomson’s gazelles with hidden fawns. Anim. Behav..

[58] Montero-Quintana A.N., Vazquez-Haikin J.A., Merkling T., Blanchard P.B., Osorio-Beristain M. (2020). Ecotourism impacts on the behaviour of whale sharks: An experimental approach. Oryx.

[59] Gigliotti L.C., Slotow R., Sholto-Douglas C., de Vos C., Jachowski D.S. (2021). Short-term predation risk and habitat complexity influence cheetah antipredator behaviours. Anim. Behav..

[60] Sirot E., Pays O. (2011). On the dynamics of predation risk perception for a vigilant forager. J. Theor. Biol..

[61] Carbone C., Thompson W.A., Zadorina L., Rowcliffe J.M. (2003). Competition, predation risk and patterns of flock expansion in barnacle geese (*Branta leucopsis*). J. Zool. Lond..

[62] Barros M., de Souza Silva M.A., Huston J.P., Tomaz C. (2004). Multibehavioral analysis of fear and anxiety before, during, and after experimentally induced predatory stress in *Callithrix penicillate*. Pharm. Biochem. Behav..

[63] Shonfield J. (2011). The effect of familiarity on vigilance behaviour in grey squirrels. McGill Sci. Undergrad. Res. J..

[64] Beauchamp G., Ruxton G.D. (2012). Vigilance decreases with time at loafing sites in gulls (*Larus* spp.). Ethology.

[65] Mettke-Hofmann C. (2023). When to Return to Normal? Temporal Dynamics of Vigilance in Four Situations. Birds.

[66] Childress M.J., Lung M.A. (2003). Predation risk, gender and the group size effect: Does elk vigilance depend upon the behaviour of conspecifics?. Anim. Behav..

[67] Stears K., Schmitt M.H., Wilmer C.C., Shrader A.M. (2020). Mixed-species herding levels the landscape of fear. Proc. R. Soc. B..

[68] Kautz T.M., Beyer D.E., Farley Z., Fowler N.L., Kellner III K.F., Lutto A.L., Petroelje T.R., Belant J.L. (2021). American martens use vigilance and short-term avoidance to navigate a landscape of fear from fishers at artificial scavenging sites. Sci. Rep..

[69] Ruble D.B., Verschueren S., Cristescu B., Marker L.L. (2022). Rewilding Apex Predators Has Effects on Lower Trophic Levels: Cheetahs and Ungulates in a Woodland Savanna. Animals.

[70] Uchida K., Suzuki K.K., Shimamoto T., Yanagawa H., Koizumi I. (2019). Decreased vigilance or habituation to humans? Mechanisms on increased boldness in urban animals. Behav. Ecol..

[71] Favreau F.R., Pays O., Fritz H., Goulard M., Best E.C., Goldizen A.W. (2015). Predators, food and social context shape the types of vigilance exhibited by kangaroos. Anim. Behav..

[72] Clermont J., Couchoux C., Garant D., Reale D. (2017). Assessing anti-predator decisions of foraging eastern chipmunks under varying perceived risks: The effects of physical and social environments on vigilance. Behaviour.

[73] Bragato P.J., Spencer E.E., Dickman C.R., Crowther M.S., Tulloch A., Newsome T.M. (2023). Habitat but not group size or recent predator activity affect corvid collective vigilance at carcasses. Austr. Ecol..

[74] Makin D.F., Chamaille-Jammes S., Shrader A.M. (2017). Herbivores employ a suite of antipredator behaviours to minimize risk from ambush and cursorial predators. Anim. Behav..

[75] Valeix M., Fritz H., Loveridge A.J., Davidson Z., Hunt J.E., Murindagomo F., Macdonald D.W. (2009). Does the risk of encountering lions influence African herbivore behaviour at waterholes?. Behav. Ecol. Sociobiol..

[76] Parker E.J., Hill R.A., Koyama N.F. (2022). Behavioral responses to spatial variation in perceived predation risk and resource availability in an arboreal primate. Ecosphere.

[77] Valeix M., Fritz H., Matsika R., Matsvimbo F., Madzikanda H. (2007). The role of water abundance, thermoregulation, perceived predation risk and interference competition in water access by African herbivores. Afr. J. Ecol..

[78] Votto S.E., Schlesinger C., Dyer F., Caron V., Davis J. (2022). The role of fringing vegetation in supporting avian access to arid zone waterholes. Emu. Austral. Orn..

[79] Hofmann G., Mettke-Hofmann C. (2024). Watch out! High vigilance at small waterholes when alone in open trees. PLoS ONE.

[80] Kamanda M., Ndiweni V., Imbayarwo- Chikosi V.E., Muvengwi J. (2008). The impact of tourism on sable antelope (*Hippotragus niger*) vigilance behavior at artificial waterholes during the dry season in Hwange National Park. J. Sust. Dev. Afr..

[81] Periquet S., Valeix M., Loveridge A.J., Madzikanda H., Macdonald D.W., Fritz H. (2010). Individual vigilance of African herbivores while drinking: The role of immediate predation risk and context. Anim. Behav..

[82] Hall L.K., Day C.C., Westover M.D., Edgel R.J., Larsen R.T., Knight R.N. (2013). Vigilance of kit foxes at water sources: A test of competing hypotheses for a solitary carnivore subject to predation. Behav. Proc..

[83] Dostine P.L., Johnson G.C., Franklin D.C., Zhang Y., Hempel C. (2001). Seasonal use of savanna landscapes by the Gouldian finch, *Erythrura gouldiae*, in the Yinberrie Hills area, Northern Territory. Wildl. Res..

[84] Brazill-Boast J., Dessmann J.K., Davies G.T.O., Pryke S.R., Griffith S.C. (2011). Selection of breeding habitat by the endangered Gouldian Finch (*Erythrura gouldiae*) at two spatial scales. Emu.

[85] NT Government (2021). Threatened Species of the Northern Territory. https://nt.gov.au/environment/animals/threatened-animals.

[86] Brush A.H., Seifried H. (1968). Pigmentation and feather structure in genetic variants of the Gouldian finch, *Poephila gouldiae*. Auk Ornithol. Adv..

[87] Del Hoyo J., Elliott A., Christie D.A. (2010). Handbook of the Birds of the World: Weavers to New World Warblers.

[88] Brazill-Boast J., van Rooij E., Pryke S.R., Griffith S.C. (2011). Interference from long-tailed finches constrains reproduction in the endangered Gouldian finch. J. Anim. Ecol..

[89] O’Reilly A.O., Hofmann G., Mettke-Hofmann C. (2019). Gouldian finches are followers with black-headed females taking the lead. PLoS ONE.

[90] Fernandez-Juricic E., Gall M.D., Dolan T., O’Rourke C., Thomas S., Lynch J. (2011). Visual systems and vigilance behaviour of two ground-foraging avian prey species: White-crowned sparrows and California towhees. Anim. Behav..

[91] Bednekoff P.A., Lima S.L. (2005). Testing for peripheral vigilance: Do birds value what they see when not overtly vigilant?. Anim. Behav..

[92] Crosmary W.G., Makumbe P., Cote S.D., Fritz H. (2012). Vulnerability to predation and water constraints limit behavioural adjustments of ungulates in response to hunting risk. Anim. Behav..

[93] Moore B.A., Doppler M., Young J.E., Fernandez-Juricic E. (2013). Interspecific differences in the visual system and scanning behavior of three forest passerines that form heterospecific flocks. J. Comp. Physiol. A.

[94] Evans S.M., Collins J.A., Evans R., Miller S. (1985). Patterns of drinking behaviour of some Australian estrildine finches. IBIS.

[95] Heinsohn R.G. (1887). Age-dependent vigilance in winter aggregations of cooperatively breeding white-winged choughs (*Corcorax melanorhamphos*). Behav. Ecol. Sociobiol..

[96] Boukhriss J., Selmi S., Bechet A., Nouira S. (2007). Vigilance in greater flamingos wintering in Southern Tunisia: Age-dependent flock size effect. Ethology.

[97] Fernandez-Juricic E. (2012). Sensory basis of vigilance behavior in birds: Synthesis and future prospects. Behav. Proc..

[98] Baker D.J., Stillman R.A., Smart S.L., Bullock J.M., Norris K.J. (2011). Are the costs of routine vigilance avoided by granivorous foragers?. Func. Ecol..

